# Application of Left Atrial Strain Based on Cardiac Magnetic Resonance Feature Tracking in Cardiovascular Diseases

**DOI:** 10.31083/RCM48630

**Published:** 2026-06-29

**Authors:** Hui Li, Shuang Li, Yike Zhao, Lei Xu, Baiyan Zhuang

**Affiliations:** ^1^Department of Radiology, Beijing Anzhen Hospital, Capital Medical University, Beijing Institute of Heart, Lung and Blood Vessel Diseases, 100029 Beijing, China

**Keywords:** left atrial strain, cardiac magnetic resonance feature tracking, cardiovascular disease

## Abstract

The left atrium (LA) plays a crucial role in maintaining left ventricular filling. Additionally, LA function serves as a key indicator for evaluating and grading the severity of left ventricular diastolic dysfunction. Increasing evidence demonstrates that LA function and volume are vital imaging indicators for various cardiovascular conditions. However, conventional volumetric parameters have limitations. They cannot adequately depict the phase-dependent characteristics and complex dynamics of LA activity. Recently, cardiac magnetic resonance feature tracking (CMR-FT) has emerged as an effective tool for quantifying LA strain. This technique offers superior reproducibility, lacks acoustic window constraints, and provides enhanced spatial resolution. CMR-FT-derived LA strain enables the early detection of atrial mechanical injury. Furthermore, it precisely reflects atrial phasic functional changes. These capabilities offer novel insights into the diagnosis, management, prognostic stratification, and therapeutic efficacy assessment of cardiovascular diseases. This review summarizes current research advances in LA strain assessment via CMR-FT and further provides perspectives on its clinical applications.

## 1. Introduction

With the aging of the population and the development of social economy, the incidence of cardiovascular disease has gradually increased, making them a major cause of death. It is estimated that by 2020, the overall prevalence of cardiovascular diseases has reached 48.6%, with approximately 128 million people currently affected [1]. The severe disease prevention and control situation has driven continuous innovation in clinical research and assessment techniques for cardiac diseases.

Normal cardiac hemodynamics depend on the left atrium (LA) serving as an essential element. In the past, LA assessment was confined to morphological analyses based on diameter and volume measurements [2]. However, LA enlargement alone cannot fully reflect the dynamic changes occurring throughout the cardiac cycle [3]. To overcome the limitations of traditional morphological evaluation, LA strain measurement techniques have emerged. LA strain measurement using cardiac magnetic resonance feature tracking (CMR-FT) is a relatively new technique that can track the phasic function of the LA and detect subclinical cardiac dysfunction in patients with normal LA size at an early stage [4]. Additionally, LA strain assessment enables early detection of several cardiovascular diseases, thereby assisting clinicians in decision-making and improving patient outcomes [5].

Given the potential value of LA strain assessment in the diagnosis and management of cardiovascular disease, this review aims to summarize the current understanding of LA strain assessment and explore its clinical applications.

## 2. Overview of Left Atrial Phasic Function

Hemodynamic stability and left ventricular (LV) filling are largely dependent on the normal function of the LA. Its structural remodeling and functional impairment can serve as sensitive indicators for identifying LV diastolic dysfunction and grading its severity [6]. During the entire cardiac cycle, three functional phases occurring sequentially and coordinately allow the LA to convert continuous pulmonary venous return into discontinuous LV filling, thereby facilitating effective systemic blood circulation [7]. In particular, these three phases involve the mechanisms and features outlined below: (1) Reservoir phase: During isovolumic diastole and ventricular systole, the LA acts as a “blood reservoir” due to reduced LV filling pressures, which allow the reception of pulmonary venous blood flow and lead to an increase in LA volume. (2) Conduit phase: Following mitral valve opening during early ventricular diastole, this phase relies on a fleeting pressure gradient between the LV and LA for promoting passive atrial blood discharge into the ventricle. (3) Booster pump phase: Myocardial contractility, afterload, and the Frank-Starling mechanism drive active LA contraction during LV diastole, which propels residual blood into the LV and thus provides a crucial supplementary contribution to LV filling [8,9].

Specifically, the descent of the LV base during systole affects atrial compliance and relaxation. The LA reservoir function represents this process. The LA conduit function depends on atrial compliance during ventricular diastole. This function correlates closely with LV relaxation and stiffness. Lastly, the LA booster function reflects inherent atrial contractility. LV diastolic pressure, LV compliance, and venous return modulate this function [8,10]. Notably, LA functional impairment precedes LA structural remodeling. Elevated LA pressure and volume trigger sequential histological changes, including cardiomyocyte lengthening, which subsequently induce cardiomyocyte hypertrophy, myocardial fibrosis, and progressive atrial dilatation [11,12].

## 3. Cardiac Magnetic Resonance Feature Tracking

Previously, echocardiography has stood out as the principal method applied in the evaluation of LA phasic function [13]. Speckle tracking echocardiography (STE), a standard method for assessing LA strain, offers several advantages, including accessibility, non-invasiveness, cost-effectiveness, and the ability to analyze ultrasound images both during acquisition and post-storage. However, it suffers from a limited field of view in the presence of a poor acoustic window and relatively high inter-observer variability. Compared with traditional echocardiography, cardiac magnetic resonance (CMR) has advantages such as not being limited by acoustic window conditions, higher repeatability, lower intra-observer and inter-observer variability, and better tracking quality [14]. Recently, CMR-FT has been used to quantify atrial strain with higher accuracy than STE. Truong et al. [15] demonstrated that CMR-FT achieved excellent tracking performance in the study population, with good intra-observer and inter-observer agreement (Table 1). Currently, CMR is regarded as the “gold standard” imaging method for evaluating cardiac chamber morphology and function.

**Table 1.** **Strengths and limitations of speckle tracking echocardiography and cardiac magnetic resonance feature tracking for left atrial strain assessment**.

	Strengths	Limitations
Speckle tracking echocardiography	Non-invasiveness Low cost and high clinical availability Real-time imaging, the ability to analyze ultrasound images after acquisition and storage	Inter-observer variability Limited field of view Anatomical plane restrictions Operator-dependent accuracy
Cardiac Magnetic Resonance Feature Tracking	Wide field of view High repeatability and spatial resolution Lower intra-observer and inter-observer variability Better tracking quality	High cost Long scan time Multiple strict contraindications (claustrophobia, metallic implants, ability to hold breath, and arrhythmia) Lack of objective reference standards

CMR-FT technology uses cardiac phased-array receiver coils and steady-state free precession (SSFP) sequences. CMR cine images gathered through retrospective ECG gating serve as the foundation where epicardial and endocardial borders of the LA receive manual delineation by operators. Application of automatic tracking algorithms follows to record motion within the atrium over the complete cardiac cycle. Myocardial strain is then quantified using post-processing software [16].

Due to the specific orientation of atrial fibers and the thinness of the atrial wall [8], currently, the measurement of atrial longitudinal strain is mainly used, but some studies are beginning to explore the potential of measuring atrial circumferential strain or radial strain. Myocardial deformation over the full cardiac cycle finds expression through strain, a parameter free from angle dependence that denotes the ratio of length alteration [3]. A positive or negative strain value indicates stretching or contraction of the atrial wall, respectively.

Strain (S) is calculated using the formula: ΔL/L_0_, where ΔL is the change in myocardial length and L_0_ is the original length of the myocardium.

Strain rate (SR) refers to myocardial deformation over time (the speed of myocardial deformation).

**Fig. 1.**
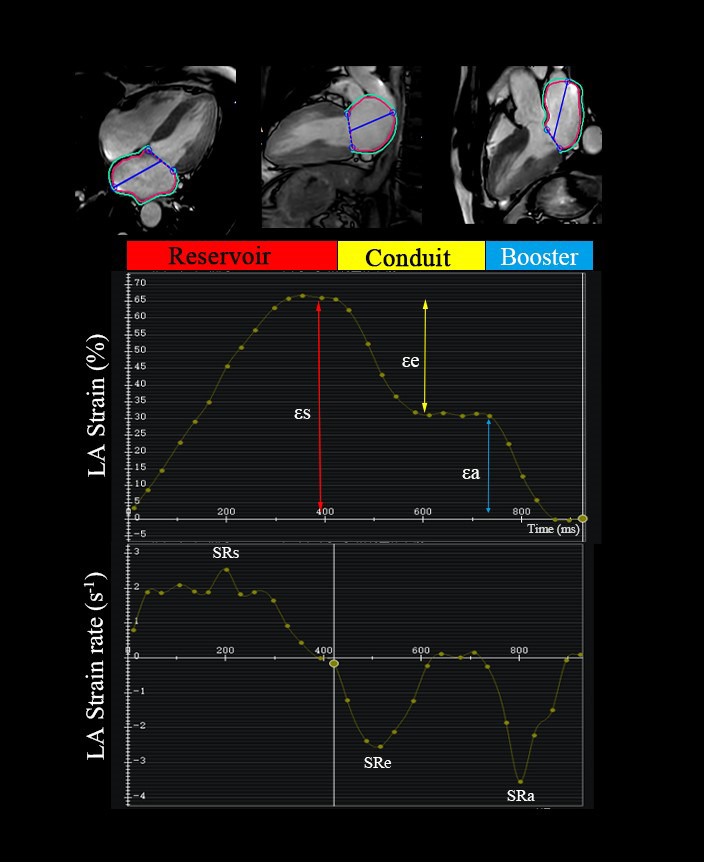
**Cardiac magnetic resonance feature tracking (CMR-FT) assessment of global peak longitudinal left atrial (LA) strain in a healthy subject via apical 3-chamber, 4-chamber, and 2-chamber views**. The lower panel shows LA strain profiles, including peak late negative strain rate (SRa), booster strain (εa), peak early negative strain rate (SRe), conduit strain (εe), reservoir strain (εs), and peak positive strain rate (SRs).

LAS and SR can quantitatively assess atrial function across three phases. The parameters mainly include reservoir strain (εs, corresponding to the atrial reservoir phase), peak positive strain rate (SRs), conduit strain (εe, corresponding to the atrial conduit phase), peak early negative strain rate (SRe), booster strain (εa, corresponding to the atrial booster phase), and peak late negative strain rate (SRa) [10] (Fig. 1).

## 4. Advances in Clinical Applications of Left Atrial Strain

This manuscript summarizes the advantages, research progress, and limitations of LA strain. Additionally, it outlines directions for future research (Table 2, Ref. [14,17,18,19,20,21,22,23,24,25,26,27,28,29,30,31,32,33,34,35,36,37,38,39,40,41,42,43,44,45,46,47,48,49,50,51,52,53,54,55]).

**Table 2.** **Clinical applications of atrial strain: prognostic value and clinical implications**.

Type of disease	Important prognostic parameters and critical values	Clinical implications of the LA strain
HCM	εs <18%: 3-fold increased risk of new-onset AF; εa <8%: 4-fold increased risk of new-onset AF [21]	Early identification of subclinical LA dysfunction and diastolic dysfunction in HCM [17,18,19]; Differentiation of HCM subtypes [18,20]; Prognostic stratification of adverse events [18,19]
DCM	εs and εe predict HF and AF [22,23,24]; εe has been identified as an independent predictor of LVRR [26]	Excellent independent prognostic indicator (superior to LV GLS and LGE) [25]
AF	εs <33%: higher risk of developing AF [54]; εa <17%: AF onset in patients with stroke risk factors [30,54]	Differentiation of AF subtypes [27,28,29]; Prediction of MACEs [30,54]; prognostic evaluation after catheter ablation [31,32,33]
MI	εs <18.8%: independent predictor of MACE following acute MI [34]; εs <19.2%: MACEs in high-risk STEMI [35]	Prognosis prediction for MACEs [34,35,36]; Assessment of LVRR [37]
HF	εs ≤15% and NT-proBNP ≥874.5 ng/L: Optimize risk stratification for adverse outcomes in HF [41]; LV GLS ≥–12.2% and LA εs ≤13.8%: Improve the accuracy of risk stratification in HFpEF [38]	Differentiation of HF phenotypes [14,39,40,55]; Prediction of adverse outcomes [41]; Early identification of high-risk groups [42]
Myocarditis	SRe = –1.6s^–1^: Distinguish AM from healthy people [43]; εs ≤25.6%: Increased risk of MACEs [46]	AM diagnosis [43]; Assessment of the degree of myocardial injury [44]; Prediction of MACEs prognosis [45,46]
CA	εs <8.6%: Increased risk of death in AL-CA patients [47]; SRe = –0.28s^–1^: Predicts all-cause mortality in AL-CA [48]	Differential diagnosis between CA and HCM [47]; Prognostic stratification of AL-CA patients [48].
CHD	εs <20%: accompanied by an increase in LA stiffness, predicts future LA dysfunction [51]	Early postoperative myocardial function monitoring [49,50]; Evaluation of intervention effects [52]
LVNC	εs: independently predicts high-risk HF [53]	High-risk HF risk prediction [53]

HCM, hypertrophic cardiomyopathy; DCM, dilated cardiomyopathy; LV GLS, left ventricular global longitudinal strain; LGE, late gadolinium enhancement; AF, atrial fibrillation; MI, myocardial infarction; STEMI, ST-segment elevation myocardial infarction; HF, heart failure; CA, cardiac amyloidosis; AL-CA, light-chain cardiac amyloidosis; CHD, congenital heart disease; LVNC, left ventricular noncompaction cardiomyopathy; MACEs, major adverse cardiovascular events; LVRR, left ventricular reverse remodeling.

### 4.1 Hypertrophic Cardiomyopathy

Hypertrophic cardiomyopathy (HCM) is one of the most common hereditary cardiovascular diseases. Its pathological features include cardiomyocyte hypertrophy, myofiber disarray, myocardial collagen proliferation, and myocardial interstitial fibrosis [17]. LV diastolic dysfunction is the predominant functional abnormality in HCM. This dysfunction steadily increases LV filling pressure. Consequently, it hinders normal blood flow from the LA to the LV. This process ultimately leads to LA structural remodeling and functional impairment [18]. LA strain allows for the accurate assessment of this functional damage. Therefore, LA strain has substantial clinical relevance for disease evaluation, adverse outcome prediction, and early lesion detection in HCM.

LA strain is an early and sensitive biomarker. It detects myocardial involvement in HCM. Compared with healthy controls (HCs), patients with HCM exhibit significant impairment in LA εe, LA εa, and LA εs [19]. Some patients with HCM present with normal LA volume and LV filling pressure. Even in these patients, LA conduit and reservoir strains are reduced [17,56]. Additionally, LA conduit and reservoir functions are impaired in patients with non-obstructive HCM (NOHCM) who have a normal LA volume index (LAVI). However, their active booster function remains preserved. This phenomenon suggests reduced LV compliance even in patients with HCM with normal LAVI. Meanwhile, the active booster pump function maintains circulatory stability through compensatory mechanisms [18]. Subsequent studies have evaluated LA strain across different patient groups. These groups include patients with hypertensive left ventricular hypertrophy (LVH), obstructive hypertrophic cardiomyopathy (OHCM), and NOHCM. Patients with OHCM exhibit more severe impairment in LA longitudinal strain. This impairment is significantly greater than in patients with hypertensive LVH or NOHCM (all *p *< 0.05). Furthermore, a significant difference in LA εs exists between patients with NOHCM and OHCM (*p* < 0.05) [20]. This establishes LA strain as an effective indicator for distinguishing HCM subtypes.

In particular, age combined with impaired LA strain serves as an independent predictor of new-onset atrial fibrillation (AF) in HCM patients: LA reservoir strain below 18% increases the risk of new-onset AF by nearly threefold, while an LA booster strain below 8% elevates this risk by almost fourfold [21]. Numerous investigations conducted across multiple centers have additionally substantiated a strong correlation linking diminished LA strain to principal cardiac results (e.g., heart failure hospitalization, cardiovascular death) within HCM patients, exhibiting prognostic merit detached from established benchmarks [17,19]. LA εe and LA εs independently predicted composite adverse events (including AF, heart failure, and cardiovascular death), with corresponding hazard ratios (HR) of 0.89 (*p* = 0.006) and 0.94 (*p* = 0.019), respectively. Incorporation LA strain parameters with LV ejection fraction (LVEF) further improved the accuracy of identifying LA-related dysfunction (AUC = 0.838) [18], thus providing a more precise basis for clinical risk stratification.

### 4.2 Dilated Cardiomyopathy

Dilated cardiomyopathy (DCM) is characterized by LV dilatation coupled with systolic dysfunction in the absence of significant coronary artery disease or abnormal loading conditions (e.g., valvular heart disease, hypertension). Relative to additional cardiomyopathies, this condition demonstrates an inferior prognosis [57]. LAS has important clinical implications in DCM for evaluating therapeutic efficacy, stratifying clinical risk, and predicting adverse events, and it serves as a key imaging parameter to guide clinical decision-making for patients with DCM.

Several studies [22,23,24] have evaluated a composite endpoint of heart failure (HF) hospitalization and all-cause mortality. These studies identify LA εe (HR = 0.91, *p* < 0.001) and LA εs (HR = 0.96, *p* < 0.001) as independent predictors of this adverse outcome. Therefore, integrating LA conduit and reservoir strains into routine clinical practice is recommended. This approach can improve risk stratification for patients with DCM.

For individuals afflicted with DCM, LAS furthermore assumes an essential function within diverse pivotal mechanisms tied to clinical judgments [58,59]. Emergence of new-onset AF amid DCM cases connects to heightened dangers encompassing stroke, mortality, and progressive HF. Independence persists for LA peak strain acting as a prognosticator regarding new AF onset among non-ischemic DCM sufferers, even following modifications to supplementary imaging and clinical factors [25].

LA conduit strain is an independent prognostic factor for left ventricular reverse remodeling (LVRR). LVRR is a key indicator of favorable clinical outcomes in DCM management. A combined model demonstrates superior prognostic value. This model incorporates the extent of late gadolinium enhancement (LGE), LA conduit strain, and New York Heart Association functional class. It achieves an area under the curve (AUC) of 0.807 (95% CI: 0.723–0.874) [26]. Therefore, this model provides an important reference for predicting therapeutic efficacy in patients with DCM. Additionally, it assists in formulating individualized treatment plans.

### 4.3 Atrial Fibrillation

Atrial fibrillation (AF) is the most common cardiac arrhythmia. LA dysfunction serves as a core pathological feature of AF and is closely associated with the incidence and mortality of cerebrovascular diseases [60]. LAS alterations exhibit significant differences across different AF subtypes and patients with comorbidities. In patients with paroxysmal AF, both LA εs and passive LA εe are significantly reduced. Moreover, LAS measured by CMR-FT can detect subtle impairments in LA booster pump function that are undetectable by the conventional active left atrial ejection fraction (LAEF) [27]. Compared with healthy subjects, patients with persistent AF exhibit significant impairment in all LA strain parameters. LA booster function is an exception. This parameter is exclusively detectable in patients with sinus rhythm [28]. In patients with AF combined with valvular heart disease, all LA strain parameters were significantly reduced (all *p* < 0.001). Additionally, total LAEF and LAS parameters in the isolated right heart valvular disease group were lower than those in the double heart valvular disease group [29].

LAS indices can predict AF onset in patients with stroke risk factors but no prior AF history—particularly, patients with LA εa <17% had a twofold increase in AF incidence [30]. This quantitative indicator provides a precise basis for screening and primary prevention of AF, enabling early clinical intervention in high-risk populations. Furthermore, LAS assessed by CMR-FT is also an important indicator for prognostic evaluation after AF catheter ablation. Habibi et al. [31] conducted a prospective longitudinal study including 51 patients with AF. These patients underwent CMR assessments at baseline, one day post-ablation, and 11 ± 2 months post-ablation. The study aimed to evaluate short-term and long-term changes in LA function following catheter ablation for AF. The results showed decreased peak LA strain, total LAEF, and LA active emptying fraction at one day post-ablation. Furthermore, total LAEF and LA strain significantly decreased during long-term follow-up in patients with AF recurrence [31]. A single-center retrospective study enrolled 52 patients with paroxysmal or persistent AF who underwent pulmonary vein isolation (PVI). Post-procedural LA function was evaluated using CMR. Impaired LA booster function reliably predicted AF recurrence at one year post-PVI (AUC = 0.73, *p* = 0.033) [32]. Another retrospective analysis included 80 patients with paroxysmal AF scheduled for catheter ablation. These patients underwent CMR imaging before and after ablation to measure LA volume and strain parameters. The analysis revealed significantly decreased LA booster strain in patients with AF recurrence post-ablation. Additionally, baseline reservoir strain, post-ablation minimal LA volume, and LA dilatation index were independently associated with AF recurrence [33]. In conclusion, these studies demonstrate that LA strain allows for the quantitative evaluation of pre-ablation LA dysfunction severity in patients with AF. Furthermore, LA strain provides essential data for post-ablation recurrence risk stratification. This promotes precise diagnostic and therapeutic strategies for AF management.

### 4.4 Myocardial Infarction

The core pathological change of myocardial infarction (MI) is the ischemic necrosis of myocardial cells. This necrosis is caused by the interruption of coronary blood supply [61]. Myocardial cell necrosis increases LV stiffness. This increase subsequently reduces LV diastolic filling and elevates LA afterload. Long-term excessive afterload triggers LA structural remodeling and functional impairment. LAS can quantitatively reflect this functional damage process. Compared with HCs, patients with STEMI exhibit significantly reduced LAS and LA SR (all *p* < 0.001) [62]. Therefore, LAS enables the early identification of LA functional abnormalities in patients with STEMI.

LAS also plays a crucial role in predicting adverse events in patients with different subtypes of MI. An LA εs under 18.8% is an independent predictor of major adverse cardiovascular events following acute MI [34]. Post-STEMI patients with an LA εs below 19.2% have a significantly higher incidence of major adverse cardiovascular events (MACEs) and are classified as high-risk. Furthermore, amalgamation of LA εs with LVEF prompts a notable AUC rise spanning from 0.713 toward 0.775 [35]. Patients encountering MACEs within a follow-up spanning 12 months exhibited substantial diminutions encompassing LA εa, LA εe, LA εs, LV global radial strain (LVGRS), LV global circumferential strain (LVGCS), and LV global longitudinal strain (LVGLS). Superiority in MACE prognostication, among such variables, pertained to LA εs which exceeded each indicator tied to LV strain [36].

In addition, LAS is an invaluable tool for assessing therapeutic efficacy by predicting LVRR after STEMI. STEMI patients who achieved LVRR showed more significant recovery of cardiac function and improvement in LA deformation. LA εs and LA εe were identified as independent predictors of LVRR [37], providing clinicians with an objective, quantitative metric to anticipate treatment response, optimize management strategies, and counsel patients on their recovery trajectory.

### 4.5 Heart Failure

Multiple etiologies contribute to heart failure (HF), which represents a syndrome exhibiting complexity and features poor prognosis together with clinical symptoms as principal characteristics [38]. Principal categorization in clinical settings presently relies upon LVEF assessment to partition HF toward three essential phenotypes: heart failure with mildly reduced ejection fraction (HFmrEF, LVEF 41–49%), heart failure with preserved ejection fraction (HFpEF, LVEF ≥50%), and heart failure with reduced ejection fraction (HFrEF, LVEF ≤40%) [63]. An impartial groundwork for differentiation across HF variants arises from LAS; it additionally operates in the capacity of a indicator linked to LA remodeling. LA conduit strain and reservoir strain demonstrated notable diminutions within individuals bearing the HFpEF phenotype (*p* < 0.01 and *p* = 0.04). Supplementary scrutiny through multivariate regression substantiated LA conduit strain functioning as the most robust prognosticator related to exercise intolerance among HFpEF phenotype carriers, indicating that it constitutes a preliminary marker associated with LA remodeling [39]. For HFrEF, Bo et al. [40] proposed that LA strain and SR were significantly lower in patients with ischemic or non-ischemic DCM combined with HFrEF, which can quantify the degree of LA functional impairment associated with cardiomyopathy. Regarding HFmrEF, Leng et al. [14] found through rapid semi-automatic analysis using CMR-FT that the impairment of LA reservoir and booster function in HFmrEF patients was significantly more severe than that in HFpEF patients. Consistent structural and functional irregularities pertaining to the LA manifested across patients bearing HFpEF, HFmrEF, and HFrEF in relation to HC cohorts, with particular demonstrations encompassing diminished LAEF, elevated minimum/maximum LA volume index, and lowered LA SR along with LA S throughout each phase. Moreover, a marked inverse association links LA phasic function to N-terminal pro-B-type natriuretic peptide (NT-proBNP). Multivariate scrutiny positions LA rapid εs in the role of a vital prognosticator concerning NT-proBNP, thus underscoring the supplementary influence exerted by LA rapid εs within HF.

Evaluating LAS is crucial for risk stratification, assessing adverse prognoses, and predicting MACEs in patients with HF. Both LA reservoir strain (AUC = 0.82) and NT-proBNP concentrations (AUC = 0.87) demonstrate strong prognostic value. They effectively predict composite adverse outcomes, including HF readmission and death. Combining an NT-proBNP level ≥874.5 ng/L with an LA reservoir strain ≤15% improves risk stratification for adverse outcomes in patients with HF. Furthermore, this combination serves as a supplementary metric. It helps guide therapeutic interventions in clinical settings [41]. Diminished LA longitudinal reservoir strain emerged within the Multi-Ethnic Study of Atherosclerosis case-control investigation as an autonomous indicator associated with HF emergence in fresh instances among multi-ethnic groups devoid of symptoms [42]. Early detection concerning cohorts facing elevated HF susceptibility finds emphasis through this, underscoring the fundamental contribution from appraising LA strain functionality. Moreover, the minimal survival devoid of events characterized patients exhibiting LAS not exceeding 13.8% in conjunction with LV GLS at or above –12.2% (Log-rank *p* < 0.001). Marked enhancement in the precision pertaining to risk categorization among HFpEF cases arises from concurrent examination involving LAS together with LV GLS via MRI [38].

### 4.6 Myocarditis

Acute myocarditis (AM) is characterized by inflammation of myocardial tissue that diverse etiologies induce, including immune abnormalities, toxins, medications, and pathogens (e.g., viruses together with bacteria) [64,65]. From an epidemiological perspective, the prevalence of AM exceeds 42% among people aged ≤35 years who die of unknown causes [66]. Additionally, with the prevalence of coronavirus disease 2019, its clinical attention has been further increased [67]. Despite the absence of a targeted therapeutic protocol aimed at AM, premature detection of involvement in the myocardium yields improved prognostic outcomes. As an indicator for quantifying myocardial mechanical function, LA strain provides a key reference for the diagnosis of AM.

LA strain holds significant diagnostic value for AM and serves as an excellent independent predictor for distinguishing AM patients from HCs. Impairment occurred in conduit strain alongside reservoir strain within the LA for those diagnosed clinically with AM. Furthermore, LA SRe emerged as the foremost standalone prognosticator concerning AM (AUC = 0.8), demonstrating a specificity reaching 80% together with sensitivity amounting to 83% [43]. Using –1.6 s^–1^ as the cut-off value for LA SRe can effectively distinguish between AM patients and healthy individuals. In a subgroup analysis of AM patients with preserved LV function, compared with the HC group, their LA SRe was significantly lower (*p* = 0.005) [68]. In addition, LA SRe and LV GLS were the optimal independent predictors of AM with preserved LV function (AUC = 0.72 and AUC = 0.69, respectively). More importantly, combining LA strain parameters with LGE or Lake Louise Criteria (AUC = 0.77) can improve the diagnostic performance of CMR for AM patients with both preserved and reduced LV function.

Substantial diminutions characterized LA SRe in the AM cohort with reduced ejection fraction alongside the one with preserved ejection fraction, as uncovered by a supplementary comparative analysis [44] relative to the control cohort. Moreover, troponin I concentrations exhibited a pronounced linkage to LA SRe, which implies potential for indirect representation of myocardial damage. Upon defining the cutoff value as –3.8 s^–1^, LA SRe yielded specificity of 92.9% combined with sensitivity of 63.9% in the differentiation between controls and AM cases featuring preserved ejection fraction (AUC = 0.822, *p* < 0.001).

Autonomous prognosticators pertaining to unfavorable clinical results among individuals afflicted with AM encompassed LA conduit strain (HR = 0.91; 95% CI: 0.84–0.98; *p *= 0.013) in conjunction with LA reservoir strain (HR = 0.90; 95% CI: 0.84–0.96; *p* = 0.002) [45]. Prognostic merit attaches to LA longitudinal strain concerning anticipation of MACE development within AM cases (AUC = 0.772, *p* < 0.05); furthermore, augmented chances of MACE incidents typify those exhibiting LA reservoir strain falling at or below 25.6% [46]. LA strain enables risk stratification for adverse clinical outcomes and MACEs in patients, thereby providing a comprehensive clinical reference for the early identification, disease severity assessment and prognostic management of AM.

### 4.7 Cardiac Amyloidosis

A collection of cardiomyopathies termed cardiac amyloidosis (CA) originates from atypical amyloid fibril buildup inside the myocardial interstitial area [69]. Progressive diastolic dysfunction accompanied by unfavorable prognosis commonly characterizes the key clinical variants, encompassing Transthyretin amyloidosis as well as Amyloid Light-Chain (AL) amyloidosis. Potential utility as diagnostic instruments for CA has undergone examination in relation to LAS metrics, with goals that include segregation from additional hypertrophic variants alongside demarcation of its clinical forms. Notable disparity proves lacking for LAS metrics when comparing individuals bearing ATTR amyloidosis to those with AL amyloidosis. Nonetheless, substantially reduced levels distinguish LAS within CA cases in contrast to observations among HCM individuals. LAS consequently assumes the function of a pivotal marker toward distinguishing HCM from CA owing to this attribute. Additionally, ECV in conjunction with LV LGE facilitated amyloid burden measurement among histologically verified AL-CA cases; outcomes revealed compromise affecting LA strain (including LA SR in addition to LAS) within those demonstrating substantial amyloid burden [47].

LAS metrics are crucial for prognostic evaluation in patients with CA. They are particularly important for predicting survival in patients with AL-CA. An LA reservoir strain below 8.6% is associated with an increased mortality risk in these patients. Furthermore, LA reservoir strain provides independent and incremental prognostic value for all-cause mortality in patients with AL-CA [47]. Recently, a retrospective study conducted a subgroup analysis by stratifying AL-CA patients into survivor and non-survivor groups based on the endpoint of all-cause mortality [48]. Compared with survivors, non-survivors exhibited significantly lower LA diastolic function parameters. These parameters included LAEF, LA longitudinal strain, and LA SRs (all *p* < 0.05). Conversely, non-survivors demonstrated a significantly higher LA volume index, LA SRe, and LA SRa. Multivariate Cox regression analysis identified LA SRe as a significant predictor of all-cause mortality (HR = 14.35; 95% CI: 1.44–142.85, *p* < 0.05). The optimal cutoff value for LA SRe was –0.28 s^–1^. Furthermore, LA SRe demonstrated incremental prognostic value (*p* < 0.05) over standard systolic indices. These indices included LV GCS, LV LGE, and LVEF. Ultimately, these findings enable more accurate risk stratification for patients with AL-CA. Additionally, they provide new perspectives for improving clinical treatment decisions.

### 4.8 Congenital Heart Disease

The pathophysiological significance and clinical value of LA function in patients with congenital heart disease (CHD) remain to be fully elucidated. Existing studies have confirmed that LA functional abnormalities can occur earlier than structural changes, which can provide important clues for postoperative condition monitoring, and also serve as a basis for postoperative management and risk stratification in CHD patients.

LAS is a sensitive imaging index. It can detect subclinical cardiac dysfunction following surgical procedures. Patients with repaired Tetralogy of Fallot (rTOF) exhibit significantly reduced LAS and SR. This reduction occurs prior to the emergence of LA enlargement. Therefore, impaired LA function represents a sensitive sign. It indicates early myocardial mechanical abnormalities following rTOF intervention [49]. Additionally, Ma et al. [50] investigated patients with repaired pulmonary atresia and ventricular septal defect. These patients demonstrated significant changes in LA function. Notably, these changes occurred despite a preserved biventricular ejection fraction. This early LA functional abnormality may be a precursor to LV diastolic dysfunction. It provides a potential marker for the early identification of postoperative LV diastolic dysfunction in patients with CHD. Furthermore, postoperative patients with CHD exhibit significantly higher LA stiffness compared with healthy individuals. Notably, all patients with an LA reservoir strain <20% demonstrated increased LA stiffness. This threshold can serve as a key indicator for predicting future LA functional impairment [51]. This enables clinicians to identify high-risk patients at an early stage and thus implement timely intervention measures.

Evaluation of intervention impacts revealed substantial improvement of LA function among rTOF patients following transcatheter pulmonary valve replacement. Evidence for this enhancement arose from prominent elevations involving LA booster strain (*p* = 0.006), conduit strain (*p* = 0.001), and reservoir strain (*p* = 0.003) [52]. The presented observations suggest that LA strain enables appraisal of early diastolic together with systolic function among CHD patients, which not merely illustrates atrial modulation concerning comprehensive cardiac diastolic filling but equally supplies critical support toward risk stratification together with postoperative management in the CHD population.

### 4.9 Left Ventricular Noncompaction Cardiomyopathy

Left ventricular noncompaction (LVNC) is an uncommon inherited cardiomyopathy. It manifests distinctive anatomical features. These features include deep intertrabecular recesses linked to the ventricular cavity. Additionally, it presents a substantially thinned compact epicardial layer and excessively proliferated trabeculae in the LV [70]. Cardiovascular mortality and morbidity exhibit a pronounced linkage with LVNC, which commonly induces dysfunction affecting LV diastole along with systole [71].

The limitations of conventional LA morphological assessments are overcome by LAS, which enables accurate identification of occult LA dysfunction in patients with LVNC, even in the absence of overt LA structural changes. LAEF in conjunction with LA longitudinal strain experiences declines among LVNC individuals, irrespective of LA expansion occurrence [53]. Subsequent to modifications encompassing LAEF, LAVI, functional and structural variables, plus clinical elements, independence marked LA εs serving as a prognosticator for high-risk HF within LVNC sufferers (HR = 23.208, 95% CI: 2.993–179.967). Patients with reduced LA longitudinal strain, decreased LAEF, and elevated LAVI exhibit an increased risk of high-risk HF events. These parameters serve as prognostic metrics for patients with LVNC. Additionally, LA εs demonstrates significant prognostic value in cases of LVNC. This finding further supports the clinical application of LA strain assessment across different cardiomyopathies.

## 5. Limitations

LA strain analysis using CMR-FT technology primarily involves single-center studies focusing on AF, HF, and cardiomyopathies. However, this technology has several limitations. Delineation of the LA endocardial border is challenging due to structural characteristics including the pulmonary veins, thin atrial wall, and LA appendage. Furthermore, strain measurements vary depending on the imaging techniques, methodologies, and software versions [3]. A previous study compared atrial strain quantifications using STE and CMR. This comparison utilized post-processing tools from Medis and CVI42. The findings indicated significantly higher LA conduit and reservoir strain values with CVI42 compared with Medis [72]. Currently, these methods lack objective reference standards and exhibit high inter-observer variability. Therefore, they require rigorous validation before widespread application. Consequently, caution is necessary when applying normal reference values in clinical practice.

Inherent limitations of CMR include multiple contraindications, long scan times, extended post-processing periods, and high costs. To overcome long post-processing times, previous studies developed a semi-automated technique. This technique quantifies LA strain and strain rate [14]. Specifically, this approach is a rapid long-axis method based on cine SSFP MRI that only requires the LA posterior wall and the midpoints of two atrioventricular junctions. These landmarks are identified on standard two-chamber and four-chamber images. Compared with traditional CMR-FT measurements, this semi-automated LA strain analysis demonstrates excellent correlation and reproducibility. Furthermore, it significantly reduces the average processing time per subject.

Future research and clinical practice require targeted strategies. These strategies will promote the standardized clinical application of CMR-FT based LA strain analysis. At the research level, standardizing measurement methods is a priority. This step eliminates systematic differences among equipment manufacturers. Additionally, age- and gender-stratified normal strain reference values require establishment. Furthermore, large-scale, prospective, multicenter studies are necessary. These studies should involve long-term multiparametric follow-up and utilize equipment from various manufacturers. Such investigations will further explore the clinical value of this technique in other cardiovascular diseases. In clinical practice, using consistent imaging modalities and post-processing software for individual patients is recommended. This approach ensures the comparability of test results.

## 6. Conclusions

CMR-FT-derived LA strain is an emerging imaging parameter with excellent reproducibility. This parameter enables the early detection of LA myocardial damage. It also facilitates a comprehensive assessment of functional changes across the three LA phases. Despite certain methodological limitations, LA strain holds significant clinical relevance across various cardiovascular conditions. Specifically, it provides a quantifiable index for evaluating treatment response, acts as an independent predictor of adverse cardiovascular outcomes in various patient populations, and enables the early detection of subclinical cardiac impairment. Future multicenter studies and improved technical standardization will likely establish LA strain as a crucial tool. This tool will aid in precise diagnostic and therapeutic strategies for cardiovascular diseases. Consequently, it will improve clinical decision-making and patient prognosis.
